# Impact of Chinese Herbal Medicine on Glucolipid Metabolic Outcomes in Women with Polycystic Ovary Syndrome: A Systematic Review and Meta-Analysis

**DOI:** 10.1155/2022/3245663

**Published:** 2022-09-30

**Authors:** Jie Li, Ruqun Zheng, Zixin Lin, Fangyuan Hu, Ying Lin, Guomei Zeng, Jieni Fang, Yingyan Shen, Huiyan Tan, Mei Han, Juan Li

**Affiliations:** ^1^Department of Traditional Chinese Medicine, The First Affiliated Hospital of Guangzhou Medical University, Guangzhou, China; ^2^Guangzhou Medical University, Guangzhou, China; ^3^The First Affiliated Hospital of Guangzhou Medical University, Guangzhou, China; ^4^Beijing University of Chinese Medicine, Beijing, China

## Abstract

**Objective:**

This investigation was conducted to analyze and evaluate the impact of Chinese herbal medicine on glucolipid metabolism in women with polycystic ovary syndrome (PCOS).

**Methods:**

We used manual and computer-aided search methods, and the search scopes included Chinese databases (China National Knowledge Infrastructure, Wanfang, the China Science and Technology Journal Database, and the Chinese Biomedical Literature Database) and English databases (PubMed, Embase, Web of Science, and the Cochrane Library). We searched these eight databases for randomized controlled trials investigating the effects of Chinese herbal medicine on glucolipid metabolism in women with PCOS, with the retrieval deadline being June 2021. Two reviewers screened, selected, and extracted data and verified the results independently. The NoteExpress software was used to manage and screen the literature, the risk of bias assessment tool was used to evaluate the methodological quality of the included studies, and the RevMan 5.4 software was used for meta-analysis.

**Results:**

A total of 13 trials were included, including 825 patients with PCOS. Because the drugs used in the control group were different, we divided the results into two parts, with four trials using placebo and nine trials using metformin as the control. The results of the meta-analysis showed that fasting insulin (MD = −2.45, 95% CI = [−4.74, −0.17], *P* = 0.04), 2 h fasting plasma glucose (MD = −0.33, 95% CI = [−0.64, −0.02], *P* = 0.04), serum total cholesterol (MD = −0.38, 95% CI = [−0.58, −0.18], *P* = 0.0002), triglycerides (MD = −0.36, 95% CI = [−0.58, −0.14], *P* = 0.001), and low-density lipoprotein cholesterol (MD = −0.58, 95% CI = [−0.75, −0.41], *P* < 0.00001) were significantly improved in the Chinese herbal medicine group compared with the placebo group. In addition, compared with metformin, body mass index (MD = −1.04, 95% CI = [−1.55, −0.53], *P* < 0.0001), serum total cholesterol (MD = −0.27, 95% CI = [−0.46, −0.07] *P* = 0.007), and low-density lipoprotein cholesterol were significantly reduced (MD = −0.12, 95% CI = [−0.22, −0.02], *P* = 0.02) and high-density lipoprotein cholesterol (MD = 0.09, 95% CI = [0.02, 0.17], *P* = 0.01) was significantly improved after treatment with Chinese herbal medicine.

**Conclusion:**

Compared with the placebo group, Chinese herbal medicine had positive effects on glucolipid metabolism in women with PCOS. Chinese herbal medicine had a positive effect on lipid metabolism when the control group was metformin, but no effect on glucose metabolism. These findings need to be verified in high-quality, large-sample, randomized controlled trials in the future.

## 1. Introduction

Polycystic ovary syndrome (PCOS) is a complex and heterogeneous endocrine and metabolic disorder in reproductive-age women [1]. PCOS commonly manifests as ovulatory dysfunction, elevated androgen levels, polycystic ovaries, insulin resistance (IR), and obesity. Studies have shown that hyperandrogenism and IR are the core etiology and main endocrine features of PCOS [2]. IR not only affects the reproductive function of PCOS patients, but also significantly increases the risk of chronic metabolic diseases—such as hyperlipidemia, hyperglycemia, cardiovascular disease, and type2 diabetes [3]—and metabolic disorders are considered to be the most important long-term concerns related to PCOS [4, 5]. In addition, the healthcare system bears a huge burden for treating the direct and indirect diseases related to IR [6]. Thus, IR has become the main focus of treatment for patients with PCOS.

There are no effective curative treatments for PCOS due to its requirement for long-term treatment. At present, syndrome differentiation dictates the main clinical treatments. Lifestyle interventions such as strength training, diet, and changing poor habits (for example, giving up smoking and drinking) have positive effects in patients with PCOS and are currently the first-line treatments [7]. However, lifestyle interventions face various challenges as the first-line management, for instance, suboptimal response and lack of adherence. Therefore, patients require additional pharmacological interventions. Metformin, the most extensively studied insulin-sensitizing agent for the treatment of women with PCOS, reduces serum insulin and androgen levels and improves ovulatory function [8]. In addition, metformin can reduce low-density lipoprotein cholesterol (LDL-C) and triglyceride (TG) levels to lower the risk of complications such as cardiovascular disease [9]. However, oral metformin has a high incidence of adverse reactions such as gastrointestinal distress, nausea, vomiting, anorexia, etc., and can even lead to death [10, 11]. It is difficult for some patients to accept these possible risks, and thus metformin is not an ideal choice for long-termfirst-line medication for patients. Therefore, the use of complementary treatments has increased in recent years [10, 11].

Traditional Chinese medicine (TCM), as a form of complementary medicine, has the clinical advantages of obvious curative effects, minimal side effects, low cost, and fewer complications in the treatment of IR in PCOS patients and has gradually become a new choice for people [12]. The treatment of PCOS should be sustainable and dynamic and should be adapted to the changing circumstances and expectations of the individual patient. In addition, patients with PCOS have different clinical symptoms, and TCM is amenable to individualized treatments. Doctors can analyze the physical condition of patients with PCOS and then formulate corresponding treatment plans. Owing to the lack of efficacy and the debilitating side effects of pharmaceuticals, complementary and alternative drugs are becoming more and more popular in the treatment of PCOS [13]. Clinical studies have further shown that Chinese herbal medicine has a definite effect in regulating glucolipid metabolism disorders [14, 15], and numerous experimental studies have found that Chinese herbal medicine also has the effect of improving glucolipid metabolism in animal models of PCOS and IR [16–18]. Overall, the available evidence suggests that Chinese herbal medicine has higher efficacy and safety for patients with PCOS compared to pharmaceutical treatments.

However, the effects of Chinese herbal medicine on glucolipid metabolism in PCOS patients have not been comprehensively analyzed and studied. Therefore, on the basis of the existing evidence, we conducted a comprehensive search of domestic and foreign literature to objectively evaluate the clinical efficacy and safety of Chinese herbal medicine on glucolipid metabolism in PCOS patients, aiming to provide the latest basis for clinical medication.

## 2. Methods and Materials

### 2.1. PRISMAP

Preferred Reporting Items for Systematic Reviews and Meta-Analyses Protocols (PRISMAP) was followed for the systematic review and meta-analysis.

### 2.2. Search Strategy

Two reviewers conducted a systematic literature search in English databases (PubMed, Embase, Web of Science, and the Cochrane Library) and Chinese databases (China National Knowledge Infrastructure (CNKI), Wanfang, the China Science and Technology Journal Database (VIP), and the Chinese Biomedical Literature Database (CBM)). Search terms were related to Chinese herbal medicine (e.g., “Oriental medicine,” “East Asian medicine,” and “Chinese herbal drugs”), PCOS (e.g., “polycystic ovary syndrome”), glucolipid metabolism (e.g., “insulin sensitivity,” “glucose tolerance tests,” “lipid profile,” “HbA1c,” “triglycerides,” “total cholesterol,” “high-density lipoprotein cholesterol,” and “low-density lipoprotein cholesterol”), and randomized controlled trials (e.g., “clinical trial,” “RCT,” “random,” “randomize,” and “randomization”). Both text and MeSH terms were used. We searched all the above databases until June 22, 2021, and two reviewers screened, selected, and extracted data and cross-verified the results of the data extraction independently.

### 2.3. Eligibility Criteria

The PICOS (population, intervention, comparison, results, and study design) framework was used to establish the selection criteria.

#### 2.3.1. Types of Studies

We only included randomized controlled trials (RCTs) in Chinese or English that used Chinese herbal medicine to treat PCOS patients. The status or date of the study had no effect on the systematic review.

#### 2.3.2. Participants

Participants in all included RCTs were adult patients with PCOS, and all participants were diagnosed using the 2003 Rotterdam criteria [19]. This definition proposes that PCOS can be diagnosed in any woman presenting with at least two of the three following criteria: hyperandrogenism (either clinical or hyperandrogenemia), ovulation dysfunction, and polycystic ovaries on ultrasound plus the exclusion of other diagnoses that could result in hyperandrogenism or ovulatory dysfunction. The participants were not excluded by their race, background, or body size, but participants with other serious diseases (such as cancer, liver disease, or kidney disease) were excluded from the RCTs.

#### 2.3.3. Intervention Groups

The included RCTs used various forms of Chinese herbal medicine treatment, including Chinese herbal decoctions or proprietary Chinese medicines derived from botanicals, minerals, animals, or chemicals. The dosage forms of Chinese medicine included decoctions, tablets, powders, pills, granules, capsules, ointments, oral liquids, plasters, and injections. Nonherbal interventions (such as massage, acupuncture, cupping, and other TCM treatments), herbal injections, or combined interventions using two or more different types of herbal medicines were excluded from the included RCTs. There were no restrictions on the herbal composition, dosage, frequency of intake, or duration of treatment in the included RCTs.

#### 2.3.4. Comparison Groups

Patients in the control groups received Western medicine (including insulin sensitizers such as metformin), placebo, Western medicine combined with placebo, or lifestyle management, including weight loss through diet and exercise. There were no restrictions on the dosage form, quantity, or duration of the medicine or placebo.

#### 2.3.5. Outcome Measures

The primary outcome was homeostatic model assessment of insulin resistance (HOMA-IR), and the secondary outcomes were fasting blood glucose (FPG), fasting plasma insulin (FINS), 2-hour fasting blood glucose (2hFPG), 2-hour fasting insulin (2hFINS), serum total cholesterol (TC), TG, high-density lipoprotein cholesterol (HDL-C), LDL-C, body mass index (BMI), and waist-to-hip ratio (WHR). The safety indicator was any adverse event.

### 2.4. Literature Screening

The identified articles were initially imported into NoteExpress. After reading the title and abstract, initial screening was performed according to the inclusion criteria. The full texts of the qualifying trials were then read to check whether the papers met the aforementioned inclusion criteria. All duplicate trials were excluded. If there was any disagreement between the two researchers, a third researcher was consulted.

### 2.5. Data Extraction and Management

The following data were collected from each study: (a) Year of publication; (b) Name of first author; (c) Country; (d) Basic characteristics of the included patients; (e) Sample size; (f) Intervention measures; (g) Outcome measures; (h) Adverse reactions; (i) Random allocation methods; and (j) Other relevant information. Microsoft Excel was used for data extraction. If there was any disagreement between the two researchers, a third researcher was consulted.

### 2.6. Bias Risk Assessment of the Included Studies

Two researchers independently assessed the quality of the literature by using the Cochrane collaborative bias risk assessment tool. The evaluation was conducted according to the standards proposed in the Cochrane Intervention System Evaluation Manual, and the risk of bias was divided into three levels of low, high, and unclear. The bias risk assessment included the following seven aspects: random sequence generation, allocation concealment, blinding of participants and personnel, blinding of outcome assessment, incomplete outcome data, selective reporting, and other bias. If there was any disagreement between the two researchers, a third researcher was consulted.

### 2.7. Data Synthesis

Data analysis was performed with the RevMan 5.4 software. When the included studies used the same measurement scale, the mean difference (MD) and 95% confidence interval (CI) were used to describe continuous variables. Because the prescriptions used in TCM are different and vary from person to person, there is inevitably heterogeneity in clinical indicators. Therefore, in this meta-analysis, the random-effects model was adopted uniformly.

## 3. Results

### 3.1. Literature Search and Screening Flowchart

**Figure 1:**
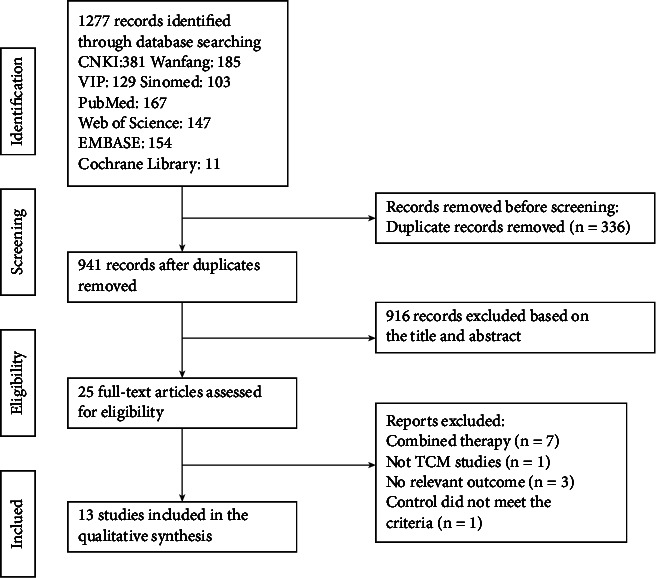
Literature search and screening flowchart.

The selection of the studies is shown in Figure 1. A total of 1277 trials were retrieved in the initial search. After deleting duplicates and filtering through the titles and abstracts, we obtained 25 full texts. Finally, 13 studies were included in the systematic review and meta-analysis [20–32].

### 3.2. Features of the Included Trials

A total of 825 patients were included in the 13 RCTs. All participants were diagnosed with PCOS according to the Rotterdam criteria, and they only received Chinese herbal medicine treatment or treatment with placebo or metformin. The specific characteristics of these studies are summarized in Table 1, including author, year, country, sample size, age, intervention measures, duration of treatment, and outcome indicators, and the main ingredients and usages of Chinese herbal medicine are summarized in Table 2.

### 3.3. Evaluation of the Quality of the Trials

**Figure 2:**
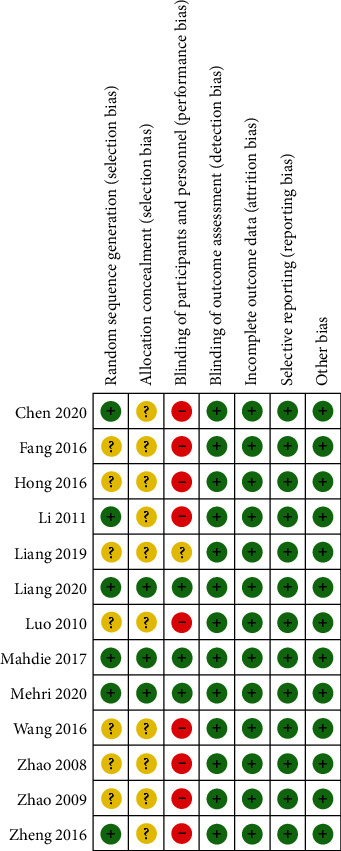
Evaluation of the risk biases of the included studies.

Cochrane collaborative bias risk assessment was used to evaluate the quality of the literature. Based on standards outlined in the Cochrane Intervention System Evaluation Manual, two researchers independently assessed the literature. The risk of bias of these 13 trials was divided into three levels of low, high, and unclear. All 13 trials mentioned the word “random” or “randomization,” and six studies described the specific randomization methods, of which only three studies described both allocation concealment and randomization methods. For participant and personnel blinding, three trials had a low risk of bias [28, 29, 32], one study did not have enough information to judge the risk level [31], and the remaining studies were at high risk of bias due to the lack of blinding during implementation. Measurements were generally made by third parties other than the researchers, so the blinding of outcome assessment was defined as low risk. In addition, all studies described the missing data and reported both the glucose metabolism and lipid profile indicators. Detailed information of the quality evaluation is shown in Figure 2. If there was any discrepancy between the two researchers, a third researcher was consulted.

### 3.4. Results of the Meta-Analysis

#### 3.4.1. Compared with the Placebo Group

*(1) General Indicators*.

**Figure 3:**
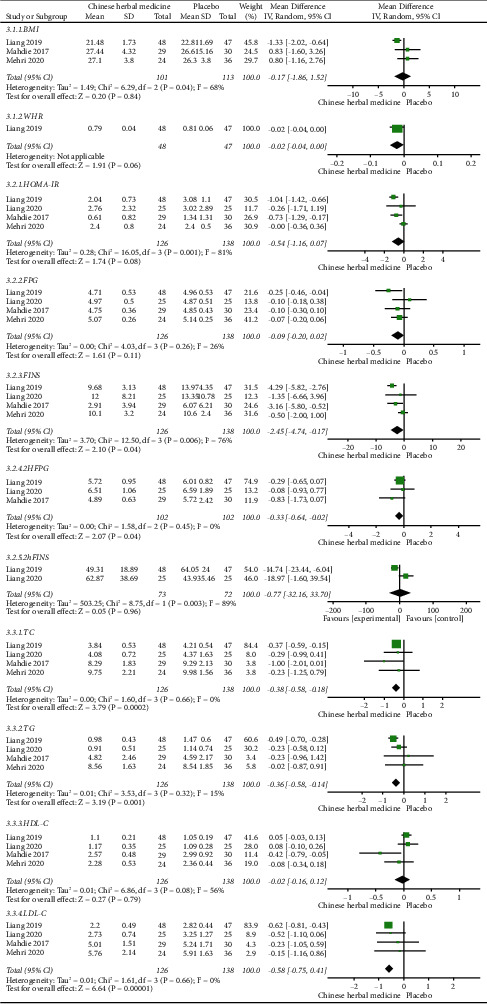
Comparisons of glucolipid metabolism between the Chinese herbal medicine group and placebo group in the treatment of PCOS.

Meta-analysis showed no statistically significant differences for BMI (*P* = 0.84) or WHR (*P* = 0.06) between the Chinese herbal medicine groups and the placebo groups. The results of the meta-analysis are shown in Figure 3 (3.1.1–3.1.2).

*(2) Glucose Metabolism Indicators*.

The analysis showed that compared with the placebo group, FINS (MD = −2.45, 95% CI = [−4.74, −0.17], *P* = 0.04) and 2hFPG (MD = −0.33, 95% CI = [−0.64, −0.02], *P* = 0.04) were significantly improved in the Chinese herbal medicine group. However, no significant difference was seen in the HOMA-IR (*P* = 0.08), FPG (*P* = 0.11), or 2hFINS (*P* = 0.96) between the two groups. The results of the meta-analysis are shown in Figure 3 (3.2.1–3.2.5).

*(3) Lipid Profile Indicators*.

The analysis showed that the indicators of TC (MD = −0.38, 95% CI = [−0.58, −0.18], *P* = 0.0002), TG (MD = −0.36, 95% CI = [−0.58, −0.14], *P* = 0.001), and LDL-C (MD =−0.58, 95% CI = [−0.75, −0.41], *P* < 0.00001) were statistically different when compared with the placebo group, but there was no statistical difference in HDL-C (*P* = 0.79) when the control group was placebo. The results of the meta-analysis of blood lipid metabolism are shown in Figure 3 (3.3.1–3.3.4).

#### 3.4.2. Compared with the Metformin Group

*(1) General Indicators*.

**Figure 4:**
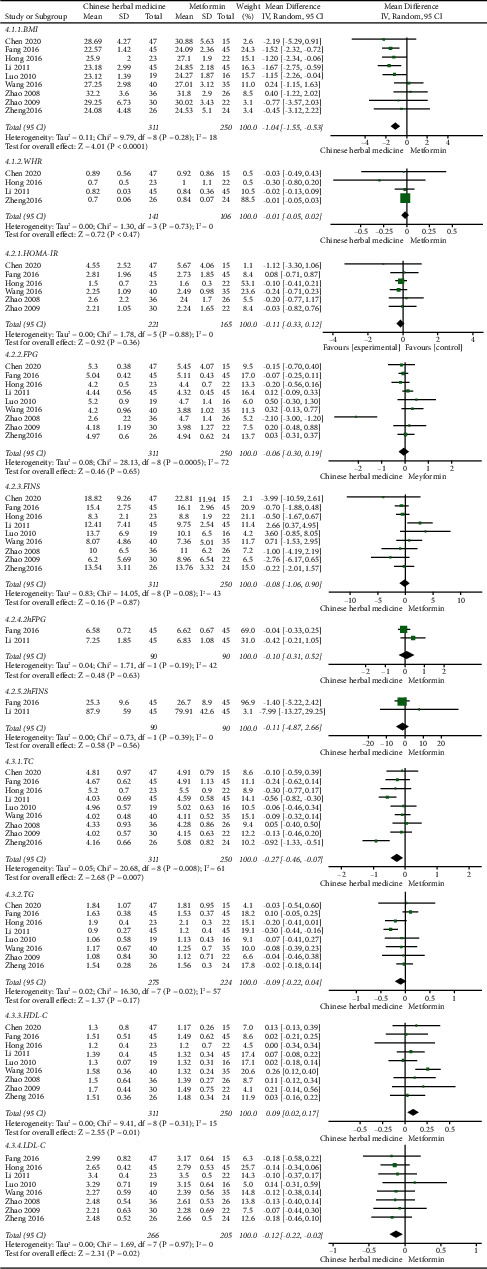
Comparisons of glucolipid metabolism between the Chinese herbal medicine and metformin group in the treatment of PCOS.

The analysis of BMI and WHR showed that compared with the metformin group, the BMI of the Chinese herbal medicine group was significantly improved (MD = −1.04, 95% CI = [−1.55, −0.53], *P* < 0.0001), while there was no statistical difference in WHR (*P* = 0.47). The results of the meta-analysis are shown in Figure 4 (4.1.1–4.1.2).

*(2) Glucose Metabolism Indicators*.

Compared with the metformin group, the analysis showed that no significant difference was seen in the HOMA-IR (*P* = 0.36), FPG (*P* = 0.65), FINS (*P* = 0.87), 2hFPG (*P* = 0.63), or 2hFINS (*P* = 0.56). The results of the meta-analysis are shown in Figure 4 (4.2.1–4.2.5).

*(3) Lipid Profile Indicators*.

The analysis showed that in the Chinese herbal medicine group, TC (MD = −0.27, 95% CI = [−0.46, −0.07], *P* = 0.007) and LDL-C (MD = −0.12, 95% CI = [−0.22, −0.02], *P* = 0.02) were significantly reduced and HDL-C (MD = 0.09, 95% CI = [0.02, 0.17], *P* = 0.01) was significantly improved. However, no significant difference was seen in the TG (*P* = 0.17). The results of the meta-analysis of blood lipid metabolism are shown in Figure 4 (4.3.1–4.3.4).

#### 3.4.3. Reporting Bias Assessment

**Figure 5:**
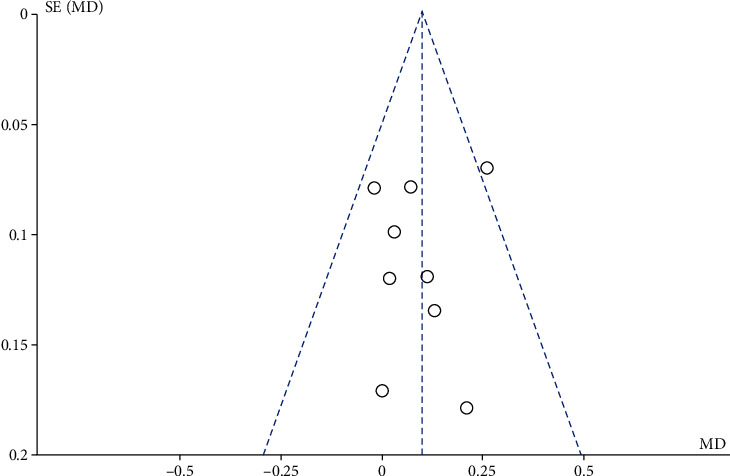
Funnel plot of comparison: HDL-C.

When more than 10 RCTs were included in the meta-analysis, a funnel plot was used to assess bias. We tried to test the indicators that included 9 RCTs of metformin as the control group in this study. The results showed that there was no obvious asymmetry in FINS and HDL-C, but the funnel plots of BMI, FPG, and TC were not symmetrical enough, suggesting that there may be publication bias. The reasons for this might be unpublished negative results, low methodological quality, or small sample sizes. They are shown in Figure 5.

#### 3.4.4. Adverse Events

Five studies reported the existence of adverse events. Three studies reported gastrointestinal side effects such as nausea, vomiting, diarrhea, or weakness in the metformin group (control group) and the patients chose to discontinue treatment [24, 26, 27]. Two of the studies reported that there were patients with abdominal distension and anorexia in the Chinese herbal medicine group, but the patients could tolerate these effects and they resolved spontaneously [26, 31]. In another study, a patient in the Chinese herbal medicine group developed skin rash and itching after using cinnamon capsules for 5 days, but the adverse effect disappeared after discontinuation of the treatment without any intervention [32].

## 4. Discussion

In this study, a meta-analysis was performed to systematically evaluate the effect of Chinese herbal medicine on glucolipid metabolism in women with PCOS. The results showed that compared with the placebo group, Chinese herbal medicine has a relatively positive effect on glucolipid metabolism in women with PCOS. Chinese herbal medicine has relatively positive effects on lipid metabolism when the control group was metformin, but had no significant effect on glucose metabolism. Overall, the effects of Chinese herbal medicine on glucose metabolism were not as significant as metformin, but were more effective than placebo. Although the results of this meta-analysis showed that insulin resistance was not significantly improved, there have still been many related reports of TCM improving insulin resistance [31, 33]. There is also basic research suggesting that Chinese herbal medicine has the potential to modulate the gut microbiota in order to control inflammation and improve insulin resistance in PCOS patients [34]. Chinese herbal medicine rarely caused adverse events, and the reported adverse events, such as abdominal distension, did not have a significant impact, indicating that Chinese herbal medicine is relatively safe and reliable for treating PCOS. Therefore, we conclude that, compared with standard treatments, Chinese herbal medicine is more effective and safer in ameliorating lipid metabolism in patients with PCOS.

However, current meta-analyses of the Chinese herbal medicine treatment of PCOS have mainly focused on infertility and obesity [35, 36], thus there is a lack of research on using Chinese herbal medicine to improve glucolipid metabolism in PCOS patients. At present, the treatments for PCOS are mainly symptomatic treatments, but Chinese herbal medicine treatment focuses on the overall health of the patient, not just their specific symptoms. In the included trials of this meta-analysis, a large percent of the prescriptions were adjusted by physicians based on clinical experience. There are also many classical prescriptions commonly used in clinical practice that were selected, such as Banxia Xiexin decoction, Cangfu Daotan decoction, and Wuji San. The compositions of the prescription are different, but most of them are mainly based on resolving phlegm and removing blood stasis. PCOS patients are often troubled by obesity, and TCM believes that the blockage of “phlegm turbidity” or “static blood” is the main pathological factor behind obesity, and thus the constitution of phlegm dampness and kidney deficiency are likely to be the important pathological basis of obesity and infertility in patients with PCOS.

Modern pharmacology has found that the active polysaccharide in Angelica sinensis has a variety of pharmacological activities, including hematopoietic activity, immune promotion, anti-inflammation, antioxidation, and liver protection. [37]. Citrinin, the active ingredient in tangerine peels, has the effect of inhibiting obesity and can also improve insulin resistance by regulating the inflammatory response caused by obesity in adipose tissue [38]. In addition, some studies have shown that after screening and network pharmacology prediction, a variety of active ingredient monomers in Cangfu Daotan Tang were found to be useful in the treatment of PCOS [39]. Among them, quercetin plays a role in regulating metabolic disorders in the treatment of PCOS [40], and kaempferol can improve insulin resistance by inhibiting the inflammatory response [41].

The results of this study also show that Chinese herbal medicine has a better effect than control interventions. In addition to herbal medicine, there are other TCM treatments such as acupuncture, cupping, and massage. Among these, acupuncture greatly improves glucolipid metabolism in patients with PCOS [42]. This meta-analysis demonstrates sufficient evidence for the effectiveness of Chinese herbal medicine in the treatment of PCOS, suggesting that more PCOS patients might choose Chinese medicine as an alternative adjuvant treatment. However, the evidence for the influence of Chinese herbal medicine on glucolipid metabolism in PCOS patients needs more high-quality, large-sample, randomized controlled trials to further confirm these conclusions.

The meta-analysis presented here improves our understanding of the therapeutic effect of Chinese herbal medicine on glucolipid metabolism in women with PCOS. However, this study also has certain limitations. First, the qualities of the included RCTs varied. Only 6 of the 13 RCTs described the specific randomization methods, of which only three studies described both randomization methods and allocation concealment, while the rest only mentioned the use of randomization methods to allocate patients, thus the accuracy and objectivity of the results are reduced. Second, the sample size of the included RCTs was small, and the clinical and physiological characteristics of PCOS were heterogeneous. Third, the choice of intervention in this meta-analysis was Chinese herbal medicine treatment, but the dosage forms, types, quantities, and courses of treatment with Chinese herbal medicine were not the same, and the treatment doses of metformin or placebo in the control groups were also different, resulting in diverse interventions in the included RCTs. Thus, our results can provide a reference for clinical practice, but further accurately designed clinical trials are needed to obtain more rigorous treatment results to overcome these shortcomings.

## 5. Conclusion

The RCTs analyzed here have shown that, compared with the placebo group, Chinese herbal medicine has relatively positive effects on glucolipid metabolism in women with PCOS. Chinese herbal medicine has a relatively positive effect on lipid metabolism when the control group is metformin, but no positive effect on glucose metabolism. Further large-scale, long-term RCTs that meet strict methodological standards are needed to prove this conclusion.

## Figures and Tables

**Table 1:** Features of the included studies.

Study ID	Sample size	Age	Treatment versus control	Duration of treatment (months)	Outcomes
Chen [23] China	T: 47	T: 28.43 ± 3.83	T: ban-xia-xie-xin-tang, decocted.	3	BMI, WHR, HOMA-IR, FPG, FINS, TC, TG, LDL-c, HDL-c
	C: 15	C: 27.27 ± 5.75	C: metformin, 500 mg, po, tid		

Fang et al. [25] China	T: 45	T:25.41 ± 3.48	T: wen-shen-tiao-jing-tang, decocted.	2	BMI, HOMA-IR, FPG, 2hFPG, FINS, 2hINS, TC, TG, HDL-c, LDL-c
	C: 45	C:25.32 ± 3.37	C: metformin, 500 mg, po, bid.		

Hong et al. [22] China	T: 23	T: 24.3 ± 5.8	T: jian-pi-qu-tan-tong-luo-tang, decocted.	3	BMI, WHR, HOMA-IR, FPG, FINS, TC, TG, HDL-c, LDL-c
	C: 22	C: 25.1 ± 6.2	C: metformin, 500 mg, po, tid.		

Li [24] China	T: 45	T: 25.33 ± 3.97	T: wu-ji-san, powdered medicine.	3	BMI, WHR, FPG, 2hFBG, FINS, 2hFINS, TC, TG, HDL-C
	C: 45	C: 25.33 ± 4.32	C: metformin, 500 mg, po, tid.		

Liang et al. [31] China	T: 48	24.69 ± 3.55	T: HYKT (Heyan Kuntai capsule)	6	BMI, WHR, HOMA-IR, FPG, 2hFBG, FINS, 2hFINS, TC, TG, HDL-C, LDL-c
	C: 47		C: placebo tid, 4 capsules each time.		

Liang [28] China	T: 25	T: 23.20 ± 3.73	T: zi-shen-qing-re-li-shi-hua-yu-fang (granules).	3	HOMA-IR, FPG, FINS, 2hFPG,2hINS, TC, TG, HDL-C, LDL-C
	C: 25	C: 24.20 ± 3.08	C: placebo granules bid, 1 bag each time.		

Luo [30] China	T: 19	T: 23.1 ± 5.7	T: bu-shen-hua-tan-qu-yu-fang, decocted.	6	BMI, FPG, FINS, T C, TG, HDL-C, LDL-C
	C: 16	C: 24.1 ± 4.3	C: metformin, 500 mg, po, qd.		

Mahdie [29] Iran	T: 29	T: 28.62 ± 5.74	T: cinnamon powder capsules	3	BMI, HOMA-IR, FPG, FINS, 2hFPG, TC, TG, LDL-C, HDL-C
	C: 30	C: 26.53 ± 6.35	C: placebo 500 mg, po, tid.		

Mehri [32] Iran	T: 24	T: 28.6 ± 4.7	T: curcumin	3	BMI, HOMA-IR, FPG, FINS, TC, TG, LDL-C, HDL-C
	C: 36	C: 27.2 ± 3.4	C: placebo 500 mg/day		

Wang et al. [20] China	T: 40	29.43 ± 4.35	T: cang-fu-dao-tan-tang modified, decocted.	3	BMI, HOMA-IR, FPG, FINS, TC, TG, HDL-C, LDL-C
	C: 35		C: metformin, 500 mg, po, tid.		

Zhao et al. [27] China	T: 36	18～35	T: yang-yin-yi-qi-huo-xue-fang, decocted.	3	BMI, HOMA-IR, FPG, FINS, TC, HDL-C, LDL- C
	C: 26		C: metformin, 500 mg, po, tid.		

Zhao et al. [26] China	T: 30	T: 23.2 ± 3.1	T: yang-yin-yi-qi-huo-xue-fang, decocted.	6	BMI, HOMA-IR, FPG, FINS, TC, TG, HDL-C, LDL-C
	C: 22	C: 24.1 ± 2.9	C: metformin, 500 mg, po, tid.		

Zheng et al. [21] China	T: 26	T: 27.02 ± 4.98	T: duo-nang-yin, decoction.	6	BMI, WHR, FPG, FINS, TC, TG, HDL-C, LDL-C
	C: 24	C: 27.45 ± 4.67	C: metformin, 500 mg, po, tid.		

T: treatment; C: control; po: peros; qd: quaque die; bid: bis in die; tid: ter in die; BMI: body mass index; WHR: waist-to-hip ratio; HOMA-IR: homeostasis model assessment of insulin resistance; FPG: fasting plasma glucose; 2hFPG: 2 h fasting plasma glucose; FINS: fasting insulin; 2hFINS: 2 h fasting insulin; TC: serum total cholesterol; TG: triglyceride; HDL-C: high-density lipoprotein cholesterol; LDL-C: low-density lipoprotein cholesterol.

**Table 2:** Ingredients and usages of Chinese herbal medicine.

Study ID	Chinese medicine decoction	Main ingredients and usage
Chen [23] China	Ban-xia-xie-xin-tang	Banxia 9 g, huangqin 30 g, huanglian 15 g, ganjiang 15 g, dangshen 12 g, shengyimi 30 g, gouqi 30 g. Add or subtract Chinese herbals according to symptoms. Decoction, 150 ml per dose. One dose each time, two times a day, once in the morning and once in the evening.

Fang et al. [25] China	Wen-shen-tiao-jing-tang	Zishiying 15 g, xianlingpi 15 g, chuanduan 10 g, tusizi 15 g, baishao 10 g, heshouwu 15 g, xiangfu 10 g, danggui 15 g, chuanniuxi 10 g, chuanjiao 10 g. Decoction in water, 1 dose a day, 250 ml per dose, orally two times, once in the morning and once in the evening.

Hong et al. [22] China	Jian-pi-qu-tan-tong-Luo-tang	Baizhu 15 g, dangshen 15 g, fuling 15 g, cangzhu 15 g, xiangfu 15 g, xianglingpi 15 g, guizhi 10 g, chaihu 10 g, danggui 15 g, taoren 10 g, shudihuang 15 g, jixueteng 15 g, chenpi 10 g. Decoction, 200 ml, 1 dose/time, 2 times/day.

Li [24] China	Wu-ji-san, powdered medicine.	Baizhi 9 g, chuanxiong 9 g, gancao 9 g, fuling 9 g, danggui 9 g, rougui 9 g, baishao 9 g, jiangbanxia 9 g, shengmahuang 9 g, chenpi 18 g, zhiqiao 18 g, cangzhu 30 g, ganjiang 10 g, jiegeng 12 g, houpo 12 g. Dissolve the medicine powder with 300 ml warm boiled water and take it two times a day, once in the morning and once in the afternoon. Do not stop taking the medicine during menstruation.

Liang et al. [31] China	HYKT (Heyan Kuntai capsule)	Dihuang, huanglian, baishao, huangqin, ejiao, fuling. 3 times a day, 4 capsules each time.

Liang [28] China	Zi-shen-qing-re-li-shi-hua-yu-fang (granules).	Zhimu 10 g, shanzhuyu 10 g, danshen 10 g, taoren 10 g, yiyiren 15 g, baijiezi 10 g, huangbai 10 g, xuanshen 10 g, gancao 6 g. Boil 1 sachet in water two times a day, take in the morning and evening, drink while still warm.

Luo [30] China	Bu-shen-hua-tan-qu-yu-fang	Tusizi 15 g, xianlingpi 15 g, roucongrong 15 g, shengdi 15 g, danggui 12 g, chuanxiong 6 g, zelan 15 g, fuling 15 g, fabanxia 10 g, cubiejia 12 g, zaojiaoci 15 g, gancao 6 g. Take one dose a day, decoct to 300 ml, and take it twice per day, once in the morning and once in the evening. Stop taking during menstruation.

Wanget al. [20] China	Cang-fu-dao-tan-tang modified	Cangzhu 12 g, xiangfu 12 g, chenpi 15 g, fabanxia 15 g, zaojiaoci 15 g, danggui 12 g, chuanxiong 12 g, shichangpu 12 g, fuling 20 g, danshen 30 g, heye 15 g, shanyao 15 g, huangqi 30 g, xianlingpi 15 g, lujiaoshuang 12 g, zishiying 30 g, sharen 6 g, chuanniuxi 15 g. Take 1 dose a day. Decoct in water two times, drink while warm, once in the morning and once in the evening. Start taking it from the 10th day of menstruation and stop taking it during menstruation, and continue for 3 consecutive months.

Zhao et al. [27] China	Yang-yin-yi-qi-huo-xue-fang	Gouqizi 10 g, ejiaozhu 10 g, zhihuangjing 10 g, hanliancao 10 g, buguzhi 10 g, nvzhenzi 10 g, digupi 10 g, maidong 10 g, nanshashen 10 g, beishashen 10 g, ziheche 10 g, shengdi 10 g, shengbaishao 10 g, huangqi 30 g, gancao 6 g. Add or subtract Chinese herbs according to menstrual symptoms.

Zhao et al. [26] China	Yang-yin-yi-qi-huo-xue-fang	Ejiaozhu 10 g, huangqin 10 g, huangbo 10 g, hanliancao 10 g, buguzhi 10 g, nvzhenzi 10 g, digupi 10 g, maidong 10 g, nanshashen 10 g, beishashen 10 g, shengbaishao 10 g, huangqi 30 g, gancao 6 g, ziheche 3 g. Add or subtract Chinese herbs according to menstrual symptoms.

Zheng [21] China	Duo-nang-yin	Tusizi 15 g, bajitian 10 g, chaihu 10 g, yinyanghuo 10 g, baishao 10 g, longdancao 6 g. Add or subtract Chinese herbs according to symptoms. Soak the herbs in 300 ml of water for 30 minutes, then boil with high heat and then simmer for 20 minutes. Take 200 ml of the first decoction, then add 200 ml of water and continue to decoct for 15 minutes. Take 150 ml of the second decoction, mix with the first 200 ml, and divide it into two doses. Take each dose in the morning and evening.

## Data Availability

This is an open-access article distributed under the Creative Commons Attribution License, which permits unrestricted use, distribution, and reproduction in any medium, provided the original work is properly cited.
